# Thrombolysis of incidental pulmonary embolism in a stroke patient

**DOI:** 10.1016/j.radcr.2024.03.053

**Published:** 2024-04-13

**Authors:** Patrick Silveira, Justin McCloskey, Mohammad Kassar

**Affiliations:** Department of Radiology, West Virginia University Hospital, 1 Medical Center Drive, PO Box 9235 Morgantown, WV, USA

**Keywords:** Pulmonary embolism, Stroke, Tenecteplase, Thrombolytic therapy

## Abstract

Both acute ischemic stroke (AIS) and pulmonary embolism (PE) are major causes of morbidity and mortality, with overlapping risk factors. Incidental or silent PE therefore may be discovered during an AIS work-up. Thrombolytic therapy is considered first-line therapy for eligible patients with AIS. We present the case of an 88-year-old man with an AIS, who was incidentally found to have a PE, and then received thrombolytic therapy leading to favorable outcomes in both conditions.

## Introduction

Each year, greater than 795,000 people in the United States will have an AIS, making in it 1 of the top 3 leading causes of death and disability [1]. PE is another major public health issue due to its high mortality, affecting about 40 to 120 per 100,000 people in the general population [2]. Though AIS and PE are distinct medical emergencies, they have many risk factors in common. Therefore, patients presenting with AIS may have a concurrent silent PE.

A routine CT stroke protocol consists of a noncontrast CT brain (NCCT), CT perfusion analysis, and CT angiography (CTA) head and neck. The initial NCCT rules out intracranial hemorrhage, permitting eligible patients to receive thrombolytic therapy. CTA is performed to detect and localize occlusion and thrombosis, extending from the aortic arch to the vertex. Consequently, the upper lobes of the lungs, pulmonary vasculature, neck, and skull are acquired during the arterial phase, allowing for incidental findings; an abnormality not directly related to the indication for the exam. Though some incidental findings, like degenerative change in the skeleton, may lack clinical significance, others, such as a mass, dissection, or PE, can have a profound impact on a patient. In this case, we describe a unique presentation of incidental PE, which nearly resolved following thrombolytic therapy in an AIS patient.

## Case report

**Fig. 1:**
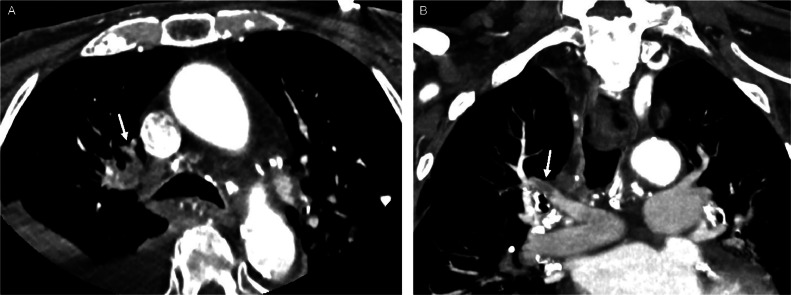
CTA stroke protocol Axial (A) and Coronal (B) images demonstrating pulmonary embolism in right upper lobe pulmonary arteries (arrow).

**Fig. 2:**
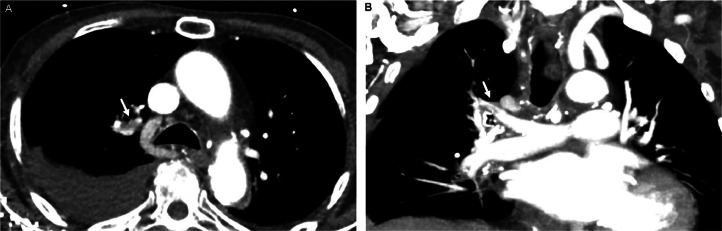
CTA chest Axial (A) and Coronal (B) images demonstrating small right sided pleural effusion and markedly decreased pulmonary embolism in right upper lobe pulmonary arteries (arrow).

An 88-year-old man with past medical history of hypertension, hyperlipidemia, diabetes mellitus type 2 presented to the emergency department with acute onset of slurred speech and hemiparesis of the right upper and lower extremities. His last known well time was 1 hour and 20 minutes prior to his arrival at the emergency department. Upon arrival, the patient was hypertensive, 150/80 mmHg, and initial labs demonstrated a blood glucose of 91 and total cholesterol of 199 mg/dl. NIH Stroke Scale/Score (NIHSS) was 9 on arrival. The patient underwent CT Stroke Protocol approximately 20 minutes after arriving to the ED, demonstrating no intracranial hemorrhage or large vessel occlusion, and received thrombolytic therapy with Tenecteplase, approximately 30 minutes after arriving to the ED; less than 3 hours since his last known well time. The patient's NIHSS 1 hour after Tenecteplase was 4, with improvement in his slurred speech and right sided hemiparesis. A pulmonary embolus of the right upper lobe pulmonary arteries was an incidental finding seen on the CTA neck (Fig.1). Approximately 24 hours later**,** CTA chest demonstrated decreased clot burden with small residual clot in the right upper lobe pulmonary artery (Fig. 2).

## Discussion

CTA is crucial for timely intervention and favorable stroke outcomes, accurately identifying acute large-vessel occlusion in a fast manner. Nevertheless, the extent of anatomy acquired in CTAs of the head and neck, combined with the urgency of an AIS diagnosis, create a situation in which critical incidental findings may be overlooked. Multiple studies have been conducted to assess the prevalence of clinically significant incidental findings on head and neck CT angiography for acute ischemic stroke (AIS). The frequently actionable findings, most commonly seen were aneurysms and pulmonary embolism [3]. Incidental PE in CTA neck was seen 0.32%-1.5% AIS patients and as high 5% in studies, when accounting for all indications of CTA neck [4], [5], [6], [7], [8]. Incidental PE has also been shown to be significantly missed in multiple retrospective studies. One retrospective study involving 1862 consecutive neck CTAs for AIS over a 2 year period found 19/19 missed incidental PE [8].

Conventional treatment of PE primarily involves anticoagulation with parenteral anticoagulants low-molecular weight heparin or unfractionated heparin and direct oral anticoagulants, rather than thrombolytic therapy. Studies have shown thrombolytic therapy to significantly reduced mortality and recurrence of PE, in high-risk PE, though evidence supporting its use in intermediate-risk PE remains controversial due to increased risk of hemorrhage [9,10]. The pulmonary embolism thrombolysis (PEITHO) trial demonstrated thrombolytic therapy with a 2.0% of hemorrhagic stroke and a 6.3% rate of major extracranial hemorrhage for intermediate-risk PE patients [11].

Tenecteplase, a genetically modified variant of alteplase, which is currently FDA-approved for acute myocardial ischemia is increasing being used off-label for AIS. Within the past five years, Tenecteplase, has become the standard of care for stroke at multiple hospital systems including the West Virginia University Health System, being cheaper, possibly safer, and more convenient to administer. Given its long half-life, tenecteplase can be conveniently administer as a single intravenous bolus over a few seconds, compared to alteplase which requires a 2-hour long infusion. It has a higher fibrin specificity compared to alteplase, meaning increased specificity in targeting the clot, potentially reducing the risk of bleeding. Studies have shown tenecteplase exhibiting greater recanalization rates than alteplase, without a corresponding increase in the occurrence of intracerebral hemorrhage. However, it is worth noting that not all clinical trials have consistently indicated the superiority of TNK over alteplase in terms of functional outcomes and early neurological improvement [12].

Multiple studies conducted on treating PE patient with tenecteplase have shown mixed results [11,13,14]. Although 1 of the more recent and largest metaanalysis of the efficacy and safety of tenecteplase on PE, showed improved 30-day survival rate without increasing hemorrhage incidents in high-risk PE and reduced risk of hemodynamic decompensation but increased risk of bleeding for intermediate-risk PE [15]. Thus, additional studies investigating the effectiveness of tenecteplase in treating PE continue.

## Conclusion

CTA in AIS patients frequently demonstrate clinically significant incidental findings such as PE that may be overlooked in a time-sensitive setting. Timely administration of thrombolytic therapy in AIS has been shown to improve patient functional outcomes. Studies looking at tenecteplase in PE has shown mixed results and are ongoing. This case demonstrates tenecteplase leading to favorable outcomes in both conditions.

## Patient consent

Informed consent was provided by the patient.
